# Failure of knee osteotomy in a case of neuropathic arthropathy of the knee

**DOI:** 10.1007/s10195-011-0137-z

**Published:** 2011-05-24

**Authors:** Francesco Traina, Marcello De Fine, Caterina Novella Abati, Ideal Frakulli, Aldo Toni

**Affiliations:** 1 1st Department of Orthopaedic Surgery, Laboratorio di Tecnologia Medica, Istituti Ortopedici Rizzoli, Via di Barbiano 1/10, 40136 Bologna, Italy; 2 Laboratorio di Tecnologia Medica, Istituto Ortopedico Rizzoli, Bologna, Italy; 3 5th Department of Orthopaedic Surgery, Istituto Ortopedico Rizzoli, Bologna, Italy

**Keywords:** Neuropathic arthropathy, Knee, Schatzker fracture, Osteotomy

## Abstract

Neuropathic arthropathy (Charcot’s joint) is a degenerative process that affects peripheral or vertebral joints as a consequence of a disturbance in proprioception and pain perception. The knee is one of the most frequently affected joints, but even when the diagnosis is made at an early stage there is no consensus on the best treatment options. An early diagnosis of neurosyphilis was made in a 55-year-old woman presenting a delayed union of an asymptomatic Schatzker type IV fracture of the proximal tibia. A medial opening wedge tibial osteotomy was performed to achieve fracture healing, to correct the medial depression of the articular surface, and possibly to avoid an early arthritis typical of the disease. To our knowledge, a knee osteotomy has never been reported at an early stage of neuropathic arthropathy. Even though the clinical and radiographic evaluation performed at 4 months follow-up showed a good stage of healing of the osteotomy and no typical features of neuropathic joint degeneration, at 8 months follow-up the knee was markedly deranged.

## Introduction

Neuropathic arthropathy (Charcot’s joint) is a degenerative process that affects peripheral or vertebral joints as a consequence of a disturbance in proprioception and pain perception. The aetiology of this condition is not yet clear and early diagnosis of Charcot’s joint is very difficult because clinical and radiographic features are not typical, but it is essential in order to modify the course of the disease [1, 2].

The knee is one of the most frequently affected joints [3], but even when the diagnosis is made at an early stage there is no consensus on the best treatment options. The majority of authors agree that a joint replacement is hazardous [4] and this is recognized to be especially true in cases of neurosyphilis even by those who report good results with knee replacement [5].

We present a case of knee osteotomy performed as a salvage procedure for a neuropathic arthropathy diagnosed at a relative early stage.

## Case Report

A 55-year-old woman was referred to us in January 2006 for a painless swelling of the left knee which started 5 months earlier after a major trauma that occurred while she was cycling. She had never suffered from relevant infections or orthopaedic pathologies, she was unable to walk without a crutch, and her left knee showed 5° of varus deformity, it was swollen and warm and its range of motion (ROM) was 0–100°. At clinical examination the left knee was painless, quadriceps hypotrophy was clear but no signs of relevant joint laxity were detected.

**Fig. 1:**
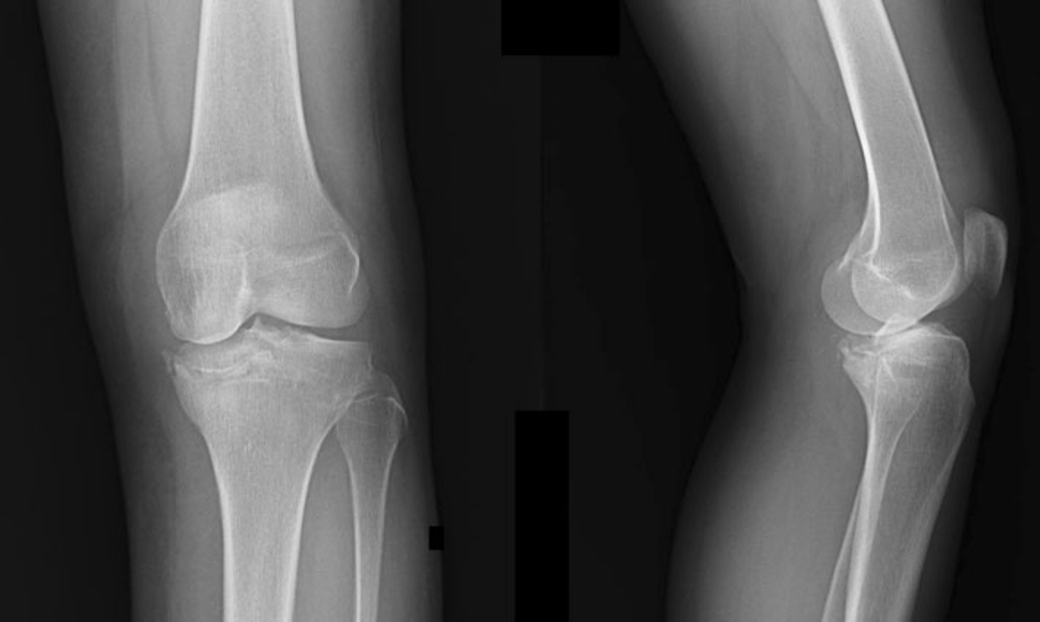
Preoperative X-rays of the left knee showing a Schatzker type IV fracture of the proximal tibia

**Fig. 2:**
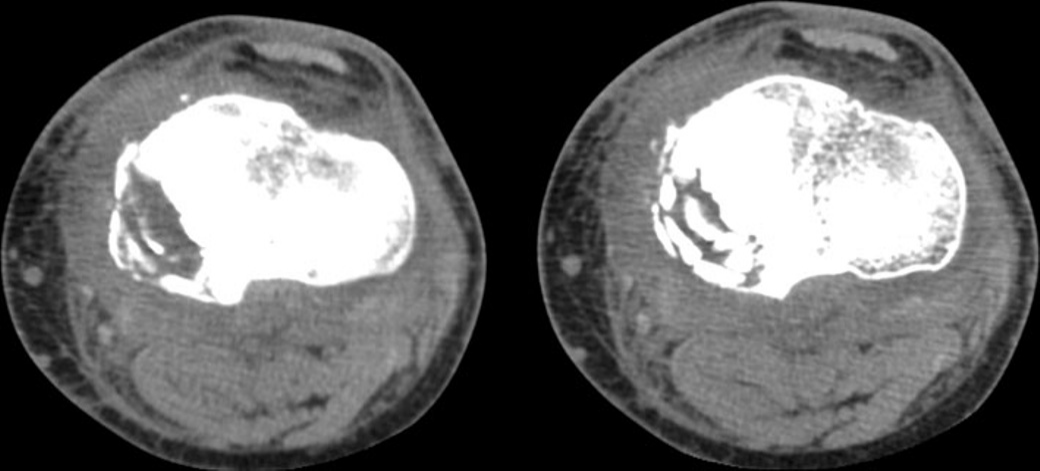
Preoperative CT scan of the left knee confirming the diagnosis of a Schatzker type IV fracture of the proximal tibia

Standard radiographs (Fig. 1) and a CT scan (Fig. 2) of the left knee revealed the delayed union of a Schatzker type IV fracture of the proximal tibia [6], probably caused by the previous trauma.

Serological tests for syphilis (VDRL-CSF, TPHA) were positive, and loss of nociception and deep tendon reflex suggested the diagnosis of neuropathic arthropathy. Benzylpenicillin (penicillin G) was administered after serological diagnosis, and ceftriaxone was added starting the day before surgery for 5 consecutive days.

A medial opening wedge tibial osteotomy was performed to achieve fracture healing, to correct the medial depression of the articular surface, and possibly to avoid an early arthritis typical of these fractures. The osteotomy was fixed with a Puddu plate and two 6.5-mm cancellous screws, and homologous bone grafting was added. Neutral alignment was achieved postoperatively.

**Fig. 3:**
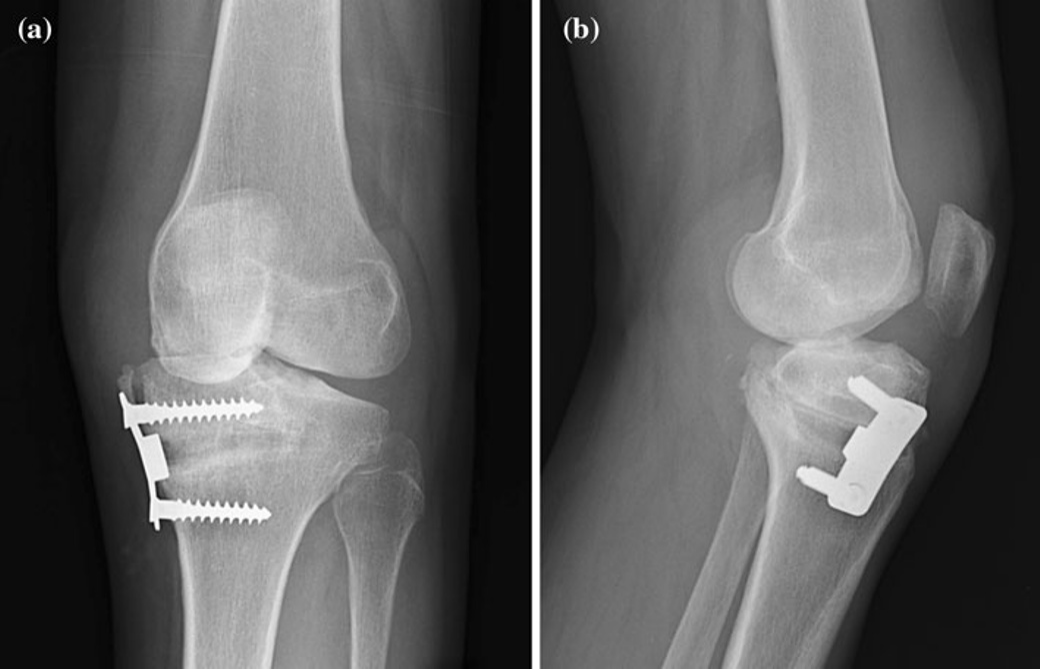
**a** Anteroposterior view of the knee at 4 months follow-up showing the good alignment of the knee and a good graft integration. **b** Lateral view of the knee at 4 months follow-up

The postoperative course was regular; at discharge weight-bearing was forbidden for 6 weeks and the use of a brace was prescribed. At 6 weeks follow-up, X-rays showed good limb alignment without loss of correction and good graft integration (Fig. 3). Weight-bearing was forbidden for two more weeks, then partial weight-bearing was allowed with the brace; physical therapy was recommended to achieve a complete ROM.

At 4 months follow-up the patient could walk using a crutch, ROM was 0–80°, and the knee was swollen. X-rays showed a partial healing of the osteotomy. Physical therapy was advised to increase quadriceps strength and ROM, and weight-bearing with the brace was allowed and encouraged.

At 6 months follow-up the patient was unable to walk without a crutch, ROM was 0–80°, and the knee was always swollen. She underwent two drainages, and culture and serological tests ruled out the hypothesis of septic arthritis.

**Fig. 4:**
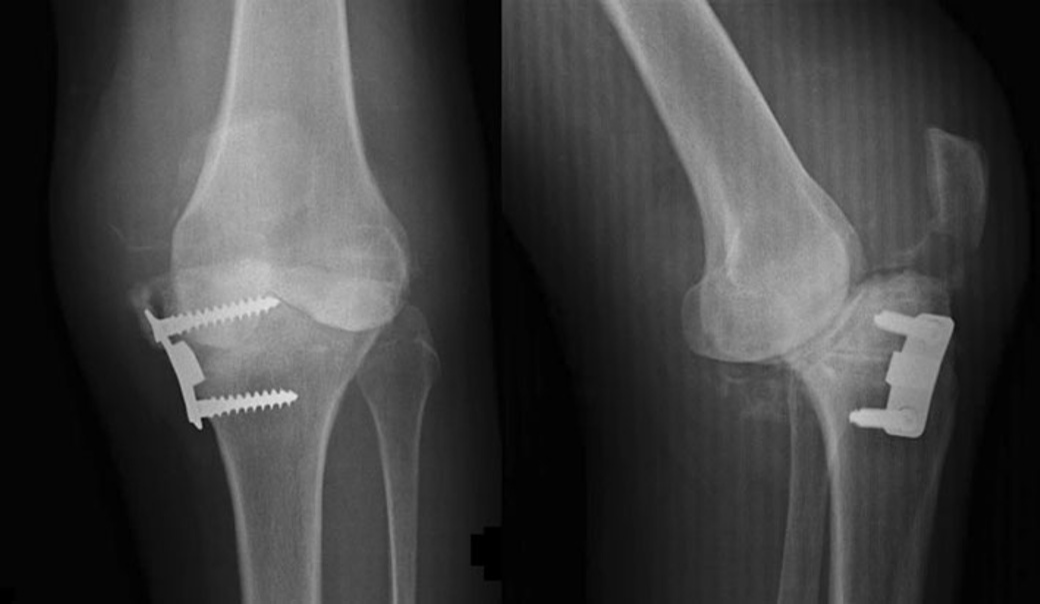
X-rays of the left knee at 8 months follow-up showing an anterior knee subluxation, osseous debris, and joint effusion

Because of the persistence of knee impairment, at 8 months follow-up she was again admitted to our institution. Standard radiographs (Fig. 4) and CT scan of the left knee showed a markedly deranged joint: there was anterior knee subluxation, calcification of soft tissues, and joint effusion. The left ankle was tender and unstable. Standard radiographs of the left ankle showed a calcaneal and fibular fracture. These findings suggested a posterior pillar pattern of disintegration of the tarsus as described by Harris [7].

The patient was discharged with advice to continue using the brace and avoid weight-bearing.

She provided an informed consent for the publication of this case report.

## Discussion

Neuropathic arthropathy has been reported in association with diabetes mellitus, syphilis, syringomyelia, congenital or traumatic spinal disorders, leprosy, congenital insensitivity to pain, Riley–Day syndrome, Charcot–Marie–Tooth disease, and many other pathologies [2].

Clinical findings in advanced stages are characteristic: the involved joint is warm, swollen, extremely unstable or even dislocated. X-rays show a grossly damaged joint and two radiographic patterns can be distinguished: the atrophic pattern, in which there is a prevalence of bone resorption that sometimes simulates a surgical amputation (especially in the upper extremity), and the hypertrophic pattern, in which there are productive phenomena, such as osteophyte formation, sclerosis and joint fragmentation. Calcification of soft tissues, debris, joint effusion, subluxation and dislocation are common both in atrophic and hypertrophic forms [9].

Unfortunately, these findings are rarely found at the beginning of a neuropathic arthropathy, and an early diagnosis is not easy.

The surgical options available to successfully treat a neuropathic arthropathy are not clearly indicated by the literature, especially in cases of neurosyphilis. Five to 10% of patients affected with tertiary syphilis develop a neuropathic arthropathy and the knee is most often involved [3].

Whereas arthrodesis seems to be the treatment of choice if performed according to the principles postulated by Drennan [10], the role of total knee arthroplasty (TKA) remains a vexed question.

Johnson claims that Charcot’s joint is a contraindication for TKA [1], while other authors report good results with this surgical procedure in neuropathic joints [5, 11, 12], especially using semi-constrained or constrained prostheses. However, all these studies present few cases and a short follow-up, thus making it impossible to draw conclusions about this matter, and a consensus on joint replacement has not been achieved [4].

At an early stage, the neuropathic arthropathy could be treated conservatively, with bracing and protective weight-bearing. These treatments can stop the vicious cycle that leads to total destruction of the insensitive joint through repeated and undetected traumas.

In our case, the patient was referred to us presenting a tibial plateau fracture with a varus deformity of the knee, a joint instability in our opinion not suitable for a conservative treatment. A high tibial osteotomy was performed in order to improve fracture healing and restore knee alignment.

Otherwise, the period of protected weight-bearing was probably too short, and in addition patient compliance was questionable for this particular treatment, and knee bracing was dismissed too early.

Nevertheless, the radiographic evaluation performed at 4 months follow up showed a good stage of healing of the osteotomy and no typical features of neuropathic joint degeneration were present (Fig. 3). The physical therapies encouraged at this stage probably caused higher stresses on the insensitive joints, causing damage to the knee and to the unprotected ankle. The osteotomy performed did not change the course of the pathology and the postoperative rehabilitation therapies probably even worsened the final outcome.

At 8 months follow-up the knee joint presented all the features of a late stage neuropathic arthropathy, leaving the patient with a serious joint impairment.

Although this case cannot be regarded as a typical case of neuropathic arthropathy since a fracture occurred complicating the course of the disease, considering the poor outcome, caution is recommended in indicating knee osteotomy for patients affected by neurosyphilis-related neuropathic arthropathy.
